# Influence of Molecular Architecture of Polycarboxylate Ether Grinding Aids on Cement Grinding Efficiency and Powder Flowability

**DOI:** 10.3390/polym18030326

**Published:** 2026-01-26

**Authors:** Yahya Kaya, Veysel Kobya, Yunus Kaya, Ali Mardani, Kambiz Ramyar

**Affiliations:** 1 Department of Civil Engineering, Faculty of Engineering, Bursa Uludag University, Bursa 16059, Turkey; 2 Department of Chemistry, Faculty of Engineering and Natural Sciences, Bursa Technical University, Bursa 16059, Turkey; 3 Department of Civil Engineering, Faculty of Engineering, Ege University, İzmir 35040, Turkey

**Keywords:** grinding efficiency, particle size distribution, powder fluidity, PCE-based GA, main chain length variation, side chain length variation

## Abstract

In this study, the effects of molecular structure parameters of polycarboxylate ether (PCE)-based grinding aids (GAs) on grinding efficiency, cement properties, and powder flowability were systematically investigated. Existing literature indicates that only limited attention has been given to a comprehensive evaluation of the combined influence of PCE molecular weight, main chain-to-side chain ratio, and side chain characteristics on the grinding process and powder behavior. Within this framework, seven different PCE-based GAs were synthesized by systematically varying the main chain length, side chain length, and side chain/main chain ratio. The structural characterization of the synthesized additives was carried out using Fourier transform infrared spectroscopy (FTIR) and gel permeation chromatography (GPC). Subsequently, the grinding efficiency, particle size distribution (PSD), and powder flowability of cements produced at two different GA dosages were evaluated in detail. The results demonstrated that increasing the GA dosage generally enhanced grinding efficiency and led to a narrower particle size distribution. An increase in main chain length at a constant side chain length improved grinding performance, whereas PCEs with a medium main chain length exhibited superior powder flowability. In contrast, increasing the side chain length alone had a limited effect on grinding efficiency. Considering all structural parameters collectively, the PCE5 additive—characterized by medium main and side chain lengths and a low side chain/main chain ratio—exhibited the most balanced and overall highest performance.

## 1. Introduction

The cement industry is increasingly criticized from a sustainability perspective due to its high energy consumption and the associated greenhouse gas emissions. Although energy-efficient production technologies and carbon capture and storage (CCS) systems have been developed to alleviate these environmental impacts, their widespread implementation remains limited by high capital and operational costs. Consequently, the development of alternative binder systems has emerged as a promising strategy to reduce the environmental footprint of cement production [1,2,3,4]. However, among the available approaches, the use of grinding aids (GAs) offers a more practical and readily applicable solution within existing cement production lines.

Grinding efficiency is influenced by both material-related and process-related parameters. Material properties such as hardness, mineralogical composition, grindability, and particle size distribution, together with operational factors including grinding speed, grinding media characteristics, feed size, and mill filling ratio, collectively govern the grinding performance [5,6].

During clinker grinding, the formation of new fracture surfaces generates highly reactive positive and negative charges. These charges promote particle agglomeration and adhesion of fine particles to the grinding media and mill internals through van der Waals forces and electrostatic interactions, thereby reducing grinding efficiency [7,8]. To mitigate these adverse effects, the incorporation of GAs into the clinker grinding process has become a common industrial practice.

Although the mechanisms by which GAs function are not yet fully elucidated, two principal mechanisms are widely discussed in the literature. The first involves a physicochemical reduction in surface energy through adsorption of the GA onto particle surfaces, while the second relates to improved particle arrangement and enhanced powder fluidity [9,10,11]. Owing to their highly polar organic structures, GAs readily adsorb onto newly formed clinker surfaces during grinding [12,13]. This adsorption alters surface energy, weakens interparticle attractive forces, and leads to a more favorable particle size distribution [12,14,15].

In recent years, amine-, glycol-, alcohol-, and carboxylic acid-based GAs have been extensively used to enhance grinding efficiency and hydration kinetics [16,17]. Nevertheless, GA dosage plays a critical role in determining not only the grinding performance but also the fresh and hardened properties of cementitious systems [6,8,14,18,19]. Moreover, the use of certain inorganic GAs at elevated dosages has been reported to affect concrete durability adversely [8]. The literature further indicates that amine-based GAs may negatively impact the rheological behavior of cementitious systems, leading to incompatibility issues with other chemical admixtures [6,14,18,19].

These limitations have driven increasing interest in next-generation polymer-based water-reducing admixtures, particularly polycarboxylate ether (PCE)-based admixtures, as potential GAs [8,20]. PCEs play a central role in modern admixture technology, as they significantly enhance workability while offering both performance and cost advantages at a constant water-to-cement ratio [21,22,23]. The global annual production of PCEs exceeding 10 million tons can be attributed mainly to the high tunability of their molecular architecture, which allows tailoring of admixture performance to specific application requirements [21,24,25].

PCEs possess a comb-like molecular structure in which the anionic main chain adsorbs onto cement particles through electrostatic interactions, while polyethylene glycol (PEG) side chains induce dispersion via steric repulsion [23,25,26]. At low water-to-cement ratios, unadsorbed PCE molecules may additionally contribute to lubrication within the system [27]. While adsorption behavior is primarily governed by the density and distribution of carboxylate groups along the main chain, steric repulsion is strongly dependent on the length, density, and flexibility of the side chains [28,29].

Due to their comparable mechanisms of action, polycarboxylate ether (PCE)-based admixtures have been increasingly explored as potential alternatives to conventional grinding aids during the clinker grinding stage [8,16,30,31,32].

Previous studies indicate that when PCEs are used at relatively high dosages, typically around 0.1% by weight of the feed, their grinding performance approaches that of traditional grinding aids, while simultaneously enhancing the dispersion and spreading behavior of cementitious systems [17,31,32,33].

Despite these findings, the literature reveals a clear gap concerning the influence of key structural parameters of PCE-based grinding aids—such as molecular weight, main chain-to-side chain ratio, and side chain characteristics—on grinding efficiency, cement properties, and powder fluidity. Although Yang et al. [17] investigated the effects of anionic charge density and side chain length on grinding performance and product characteristics, a systematic variation in these parameters and an in-depth evaluation of their combined influence on powder flow behavior remain lacking.

The molecular architecture of comb-type PCEs is inherently multi-parametric; variations in one structural feature (e.g., backbone length) may simultaneously affect related descriptors such as molecular weight, carboxylate charge density, and grafting/side-chain density. Therefore, rather than claiming fully independent one-variable-at-a-time control, the present study was designed as a targeted series comparison, where groups of PCEs were synthesized to primarily emphasize (i) backbone length, (ii) PEG side-chain length, and (iii) side-chain-to-backbone (anionic/non-ionic) ratio. This approach enables identification of dominant architectural trends in grinding efficiency and powder flowability, while acknowledging the limitations associated with co-varying parameters. The physicochemical characteristics of the synthesized admixtures were characterized using Fourier-transform infrared spectroscopy (FTIR) and gel permeation chromatography (GPC). Furthermore, the grinding efficiency, particle size distribution, and powder fluidity of the resulting cements were comprehensively evaluated using two different dosage levels of these admixtures.

## 2. Materials and Methods

### 2.1. Materials

#### 2.1.1. Synthesis Materials

Laboratory-grade sulphuric acid, mPEG with different molecular weights, polyacrylic acid, and NaOH compounds were purchased and used without purification.

#### 2.1.2. Cement Production Materials

The cements produced within the scope of the study were obtained by grinding 96% clinker and 4% gypsum in a laboratory ball mill until a target Blaine fineness of 3900 ± 100 cm^2^/g was achieved. This target Blaine value was determined based on previous research [6,19]. The cements obtained are Portland-type cements in accordance with the TS EN 197-1 Standard. In this context, some physical and chemical properties of the clinker and gypsum used are shown in Table 1.

### 2.2. Method

#### 2.2.1. Synthesis Method

**Figure 1:**
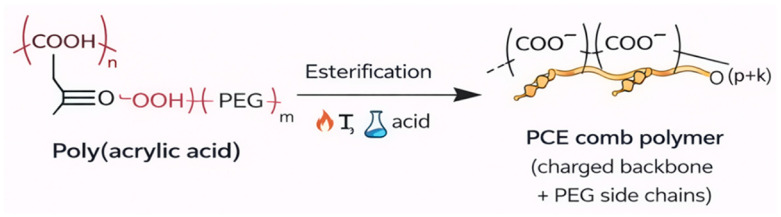
Polycarboxylate ether (PCE) synthesis scheme.

The polycarboxylate ether (PCE) used in this study was synthesized via the esterification reaction of polyacrylic acid and methyl-polyethylene glycol (mPEG) molecules with different molecular weights. The general schematic representation of the synthesized PCE molecules is provided in Figure 1.

A 500 mL three-necked reactor has a reflux condenser attached to one neck and a vacuum connection to the other. First, 100 g of mPEG was added to the reactor, which was heated to 80 °C to melt the mPEG. The reaction was initiated by adding 50 mL of polyacrylic acid and 10 mL of sulphuric acid as a catalyst. The water produced as a result of the esterification reaction was removed under vacuum to ensure that the reaction proceeded towards the products. The reaction was continued at a temperature of 150–160 °C for 4 h. The cessation of water removal under vacuum indicated the completion of the reaction. After the reaction was completed, a polycarboxylate ether polymer with different physical and chemical properties was present in the reactor. The FT-IR spectra of the obtained PCE molecules were measured using a Perkin Elmer Spectra two device (PerkinElmer, Norwalk, CT, USA). Spectral measurements were performed at a resolution of 1 cm^−1^ and with 64 scans. Thus, the molecular vibration transitions corresponding to the mid-infrared region (400–4000 cm^−1^) of all compounds and mixtures were examined, and the effect of characteristic functional groups on intermolecular interactions of the mixtures was investigated. GPC analyses were determined through service procurement.

**Figure 2:**
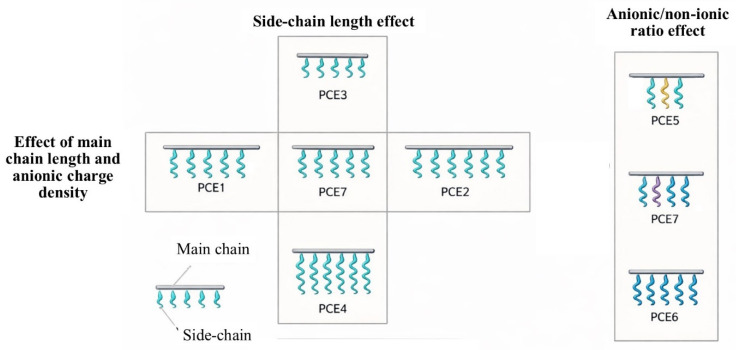
Schematic view of PCE-based GAs.

Schematic images of the synthesized PCE-based GAs according to their side chain, main chain, and other properties are depicted in Figure 2, and some of their physical and chemical properties are listed in Table 2.

To clarify the comparison logic, the synthesized PCE grinding aids were grouped into three series: (i) backbone-dominant series, in which the acrylic main chain length (and associated anionic charge density) was primarily increased while keeping the PEG type comparable; (ii) side-chain-dominant series, in which the PEG side-chain molecular weight was varied while maintaining comparable backbone characteristics; and (iii) ratio series, in which the side-chain-to-backbone ratio was adjusted to tune the balance between anionic (carboxylate) and non-ionic (PEG) contributions. The resulting polymers were characterized by FTIR and GPC, and the structural descriptors (Mn, Mw, PDI, hydrodynamic diameter) were used to interpret performance trends.

#### 2.2.2. Theoretical Calculations

In this study, structural optimization and vibrational frequency calculations of the synthesized polycarboxylate ether (PCE) polymers were performed using the Gaussian 09 software package. At the same time, GaussView 5.0 was employed for visualization and preparation of input files [34]. To represent the polymeric structure in a computationally feasible manner, model molecules consisting of a single monomer unit for each PCE chain architecture were constructed. Owing to the high molecular weight of the actual polymers, the parameters p, k, and m were fixed at unity, and the corresponding monomer units were optimized accordingly.

Following geometry optimization of the isolated PCE structures, a Ca^2+^ ion was introduced to the optimized configurations to simulate ion–polymer interactions relevant to cementitious environments, and the resulting complexes were re-optimized. All geometry optimizations were carried out using the semi-empirical PM6 method. To evaluate the adsorption behavior and interaction strength between the PCE molecules and Ca^2+^ ions, vibrational frequency calculations were subsequently performed using density functional theory (DFT) with the B3LYP functional and the 6-31G (d,p) basis set. Gibbs free energy values were obtained from these calculations to determine adsorption energies [35]. The absence of imaginary frequencies in all optimized structures confirms that the geometries correspond to true energy minima.

#### 2.2.3. Clinker Grinding Method

Clinker grinding experiments were conducted using a laboratory-scale ball mill equipped with a 1.5 kW motor and a grinding capacity of 5 kg. The mill dimensions were selected in accordance with the Bond grinding test methodology [36]. The grinding performance of PCE-based grinding aids (GAs) was evaluated by comparing the energy consumption required to achieve a target Blaine fineness value. The Blaine fineness value was determined by taking samples from three different regions of the cement produced during the grinding process.

The ball size distribution employed during grinding was determined based on preliminary optimization studies, and the standard ball charge recommended for the Bond test was adopted accordingly [6]. During the grinding process, GAs were added at dosages of 0.05% and 0.10% by weight of the clinker–gypsum mixture. The nomenclature of the resulting cement samples reflects both the type and dosage of the grinding aid; for example, cement produced using 0.05% PCE1 was designated as PCE1-0.05.

The specific energy consumption associated with the clinker grinding process was calculated using Equation (1).

E_g_ = (220 × T_g_ × A × 1000)/(m × T_d_) (1)

Here, E_g_ is the grinding energy (kWh/tonne), T_g_ is the grinding time (hours), A is the amperage, m is the feed rate (kg), and T_d_ is the mill factor (a fixed value taken as four from the manufacturer.

#### 2.2.4. Particle Size Distribution and Zeta Potential

The particle size distribution (PSD) of the ground powder samples was determined using a Malvern Mastersizer 2000 laser diffraction instrument (Malvern Panalytical, Malvern, UK) equipped with a Hydro 2000S wet dispersion unit (Malvern Panalytical, Malvern, UK). To minimize the potential errors caused by cement particles forming agglomerates and affecting the results, the samples were subjected to 10 min of ultrasonic mixing prior to measurement. The Zeta potential (ZP) measurements of the produced cements were performed using a Zetasizer ZS90 model Zetameter (Malvern Panalytical, Malvern, UK). During all ZP measurements, the temperature and voltage were maintained at 23 °C and within the range of 50–100 V, respectively [37]. Zeta potential measurements were conducted in non-aqueous media (ethanol/isopropanol) to avoid cement hydration and to better represent quasi-dry powder conditions. The cement suspensions were prepared by dispersing ground cement in alcohol and the zeta potential was determined after stabilization of the suspension.

#### 2.2.5. Calculation of the *n* Value According to the Rosin–Rammler Distribution

The Rosin–Rammler distribution is a method commonly used to express particle size distribution as a continuous function, defining particle fineness and distribution homogeneity in powdered materials. Within the scope of this study, the *n* values for the size distributions were determined using Equation (2).

(2) $$R=\exp\left[-{\left(\frac{x}{x^{1}}\right)}^{n}\right]$$

Here, R denotes the mass fraction remaining above a specific particle size; *x* is the particle size (µm); *x*^l^: characteristic size (the particle size through which 63.2% passes); *n*: represents the slope parameter of the distribution.

#### 2.2.6. Powder Flowability Experiments

In order to determine the effect of GA use on the powder properties of cement, the bulk density, loose density, Hausner ratio, Carr index, and angle of repose of cement powder were examined.

The Carr index and Hausner ratio are calculated using bulk density and loose density (Equations (3) and (4)) [38,39].

(3) $$\mathrm{CI}\;(\%)=\frac{DY-YY}{YY}\times 100$$

(4) $$\mathrm{HR}=\frac{DY}{YY}$$

Here, *DY* denotes dispersed density, and *YY* denotes bulk density.

## 3. Result and Discussion

### 3.1. Characterization of PCE-Based GAs

#### 3.1.1. FTIR Analysis

**Figure 3:**
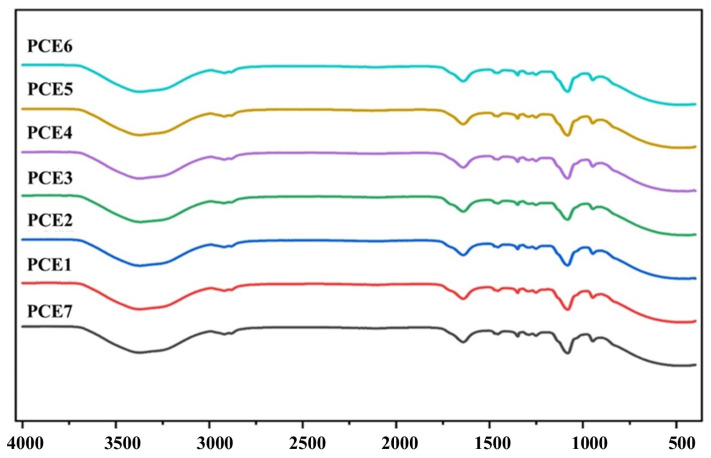
FTIR spectra of synthesized PCE-based GAs.

The FT-IR spectra of the synthesized PCE-based grinding aid molecules are presented in Figure 3. As shown in the figure, all PCE samples, despite having different main chain-to-side chain ratios, exhibit characteristic vibrational peaks at similar wavenumbers, indicating successful synthesis with comparable functional groups.

One of the most critical indicators of esterification is the carbonyl (C=O) stretching vibration. In polyacrylic acid, which is the precursor material, this vibration is observed at approximately 1734 cm^−1^. In the synthesized PCE molecules, the carbonyl stretching band shifts to lower wavenumbers in the range of 1710–1695 cm^−1^, confirming the occurrence of esterification reactions between acrylic acid and polyethylene glycol (PEG). Moreover, the integrated area of the carbonyl stretching band is expected to increase with increasing main chain density. In this respect, the PCE2 and PCE6 samples exhibit a larger carbonyl band area compared with the other PCE molecules, indicating a higher density of ester-linked main chain segments.

As observed in Figure 3, broad absorption bands with maxima at approximately 3300, 1630, and 550 cm^−1^ are attributed to stretching, in-plane bending, and out-of-plane bending vibrations associated with water molecules present in the synthesized PCE structures. These bands also include contributions from hydrogen-bonded O–H stretching and bending vibrations. The aliphatic C–H stretching vibrations of the PCE molecules are observed in the range of 2934–2877 cm^−1^, while the corresponding bending vibrations appear as weak bands between 1455 and 1253 cm^−1^.

In addition, the strong and sharp absorption band observed around 1100 cm^−1^ corresponds to the C–O stretching vibrations of the C–O–C ether groups originating from the PEG side chains. The simultaneous presence of carbonyl (C=O) and ether (C–O) stretching vibrations in the FT-IR spectra of all samples confirms that both acrylic acid and PEG monomer units are successfully incorporated into the molecular structure of the synthesized PCE-based grinding aids.

#### 3.1.2. GPC Analyses

**Figure 4:**
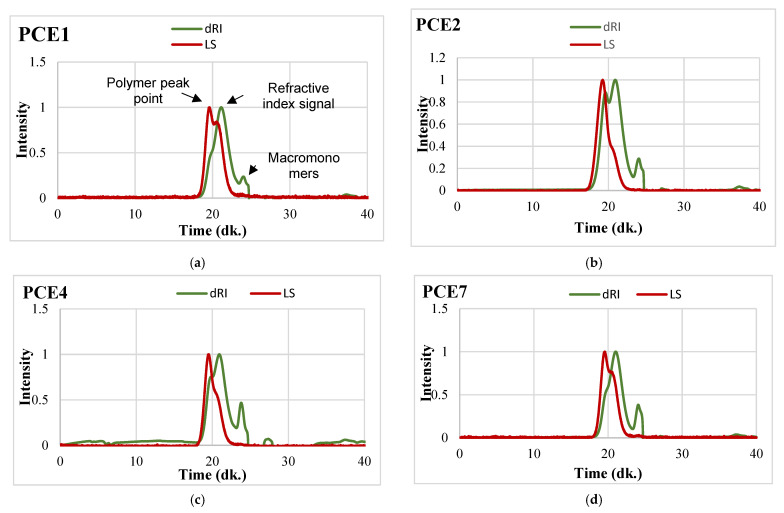
GPC analyses of some of the synthesized PCE-based GAs (**a**) PCE1 (**b**) PCE2 (**c**) PCE4, (**d**) PCE7.

GPC analyses of some of the synthesized PCE-based GAs are shown in Figure 4.

The molecular characteristics of the synthesized PCE polymers obtained from GPC analysis are presented in Figure 4 and summarized in Table 2. The results clearly indicate significant differences in molecular architecture among the PCE samples. Among the synthesized polymers, PCE2 (70 kDa) and PCE6 (69 kDa) exhibit the highest molecular weights, reflecting their long-chain structures, whereas PCE1 (24 kDa) and PCE3 (22 kDa) represent short-chain polymers with comparatively lower molecular weights.

An increase in main chain molecular weight, which is directly associated with higher carboxyl group density, was accompanied by an increase in the side chain content of the PCE molecules. Consequently, both number-average (Mn) and weight-average (Mw) molecular weights gradually increased with increasing carboxyl group density. In particular, the elevated Mn and Mw values observed for PCE2 and PCE6 can be attributed to the higher number of acrylic acid units and grafted side chains, resulting in more extended polymer backbones.

The polydispersity index (PDI) values of all PCE samples ranged between 2.0 and 2.3, indicating moderately polydisperse molecular weight distributions typical of free-radical polymerization. Among them, PCE4 exhibited the narrowest molecular weight distribution with a PDI value of 2.0, suggesting a relatively more controlled polymerization process. The side chain/main chain ratio reached its highest value in PCE2 and PCE6 (27), implying that these polymers may provide enhanced steric hindrance and solubility in aqueous environments.

Hydrodynamic diameter measurements revealed values ranging from 224 to 414 nm. PCE5 exhibited the largest hydrodynamic diameter (414 nm), indicating a more expanded molecular conformation in solution, whereas PCE2, despite its high molecular weight, showed a more compact structure with a hydrodynamic diameter of 224 nm. These results demonstrate that not only molecular weight but also chain architecture and side chain distribution strongly influence the spatial conformation and dispersion behavior of PCE polymers in solution, which in turn affect their rheological and adsorption characteristics.

### 3.2. Theoretical Calculations

To elucidate the molecular-level interaction mechanism of PCE polymers, theoretical calculations were performed using the Gaussian 09 software package. Owing to the high molecular weights of the synthesized polymers, a simplified molecular model based on the smallest repeating unit was adopted to ensure computational efficiency. Accordingly, the p, k, and m parameters were set to unity, and the monomeric unit was optimized to investigate its fundamental electronic and geometric properties. The smallest unit was designed as a model for three different monomers. It was thought that designing a larger oligomer would complicate the calculations. Although the smallest structure does not fully represent the electronic structure of the polymer molecules, it is a model created to support experimental studies. Polymer molecules are very large structures, and modeling oligomeric structures to perform the desired calculations is a common practice in the literature. While the long structure and conformational structure of the polymer chains are not fully revealed by the modeled oligomeric structure, the experimental values are supported by changes in the electronic properties of heteroatoms, which are thought to be effective in adsorption.

**Figure 5:**
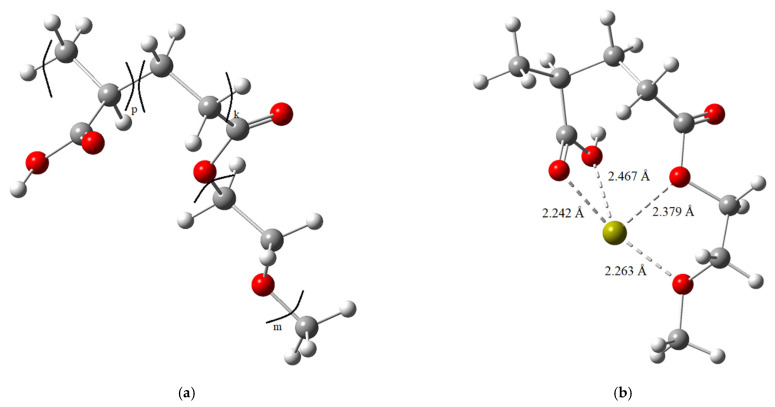
Optimized molecular structures before and after the interaction of the PCE molecule with the Ca^2+^ ion. (**a**) before; (**b**) after.

The interaction between the PCE molecule and Ca^2+^ ions, which plays a critical role in grinding efficiency and surface modification, was subsequently examined. A Ca^2+^ ion was introduced into the optimized PCE structure, and re-optimization was carried out using the same computational methodology. The optimized geometries before and after Ca^2+^ adsorption are compared in Figure 5. The results clearly show that the Ca^2+^ ion preferentially coordinates with the oxygen atoms of the PCE molecule, highlighting the strong ion–ligand interaction facilitated by the high electron density of the carboxylate and ether groups.

Energy calculations performed using the B3LYP/6-31G (d,p) method yielded an adsorption energy of −1.418 eV, indicating that the formation of the PCE–Ca^2+^ complex is thermodynamically favorable and occurs spontaneously. Moreover, the absence of imaginary frequencies in the optimized structures confirms that the system resides in a stable and energetically favorable configuration.

These theoretical findings demonstrate that PCE polymers can strongly interact with Ca^2+^ ions released from clinker surfaces, leading to effective surface adsorption and modification. Such interactions reduce electrostatic attraction and agglomeration between cement particles, thereby enhancing dispersion, improving grinding efficiency, and increasing the fluidity of the powder. Consequently, the theoretical calculations provide a robust molecular-scale explanation for the experimentally observed performance of PCE-based grinding aids in cement grinding applications.

### 3.3. Grinding Efficiency

The effect of the newly synthesized PCE-based GA on grinding efficiency was evaluated based on the energy expended to achieve the target Blaine fineness value (3900 ± 100 cm^2^/g). The results obtained from the grinding process are presented in Table 3.

**Figure 6:**
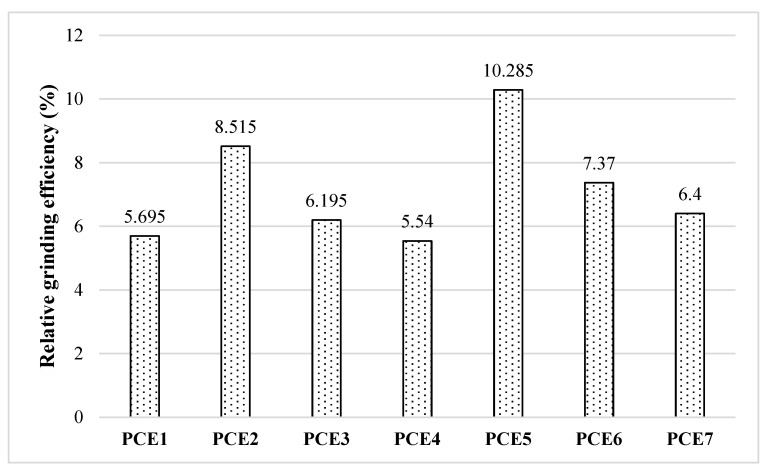
Average relative energy efficiency performance of PCE-based GAs.

The primary factors influencing the clinker grinding stage are, firstly, neutralizing surface charges by adsorbing them onto the cement surface, and secondly, optimizing cement powder fluidity to increase material capture between the balls within the mill. Regardless of GA type and dosage, the use of PCE-based grinding aids resulted in a 4–12% increase in grinding efficiency compared to the control sample. To systematically evaluate their grinding performance, the average energy efficiencies of the PCE-based GAs are presented in Table 4 and Figure 6. The grinding efficiencies of the cements incorporating PCE additives were analyzed by considering variations in anionic charge density, main chain length, side chain length, and anionic/nonionic ratio. Among the synthesized additives, PCE7—characterized by medium main chain and side chain lengths and an intermediate anionic/nonionic ratio—is included under all three parameter groupings. This approach enables a clearer assessment of the effects associated with both increasing and decreasing each structural variable.

Unlike conventional amine- or glycol-based grinding aids, which primarily function by reducing surface energy, PCEs act as structurally tunable polymeric systems capable of establishing multiple physicochemical interactions with cement particles [24,32]. During grinding, freshly fractured clinker surfaces expose high-energy, Ca^2+^-rich active sites. PCE molecules adsorb onto these sites mainly through their anionic functional groups, while their nonionic side chains extend into the surrounding space, generating steric hindrance and increasing interparticle separation. This combined electrostatic and steric stabilization mechanism governs fracture efficiency, suppresses particle agglomeration, and plays a crucial role in controlling particle size distribution (PSD) throughout the grinding process [17].

The main chain length emerges as a critical parameter controlling the continuity of adsorption and the surface coverage efficiency of PCEs on cement particles. In short, main-chain PCEs have a limited number of functional groups, which restricts the number of adsorption sites per molecule, resulting in localized and relatively transient surface interactions and a weaker stabilization effect on fine particles [22,24,25]. As the main chain length increases, the polymer can anchor to the surface through multiple interaction points, forming a more continuous and stable adsorbed layer. This enhanced surface coverage effectively inhibits the re-agglomeration of fine particles generated during grinding, resulting in increased proportions of particles smaller than 32 µm, and particularly those smaller than 10 µm [31]. However, excessive main chain length may reduce molecular flexibility or induce unfavorable conformations on the particle surface, potentially limiting PSD uniformity. Consequently, while increasing main chain length generally improves grinding efficiency, an optimum range is required to balance adsorption strength and molecular adaptability [17].

Side chain length directly determines the steric hindrance capacity of PCE molecules during grinding. Side chains protruding from the adsorbed main chain create a physical barrier that prevents close particle–particle contact. When side chains are too short, this barrier effect is insufficient, allowing fine particles to re-agglomerate through Van der Waals attractions [24,40]. Medium-length side chains provide an optimal balance by stabilizing surface adsorption while effectively increasing interparticle distance, resulting in minimized d_50_ and d_90_ values and a narrower, more controlled PSD. In contrast, excessively long side chains may introduce adverse effects such as intermolecular entanglement, irregular surface adsorption, or partial obstruction of access to fracture sites [22,24,25,41]. These effects can hinder fracture kinetics and ultimately reduce grinding efficiency. Therefore, side chain length, similar to main chain length, exhibits a well-defined optimum range for maximizing grinding performance.

The anionic/nonionic ratio represents a key structural parameter governing the balance between electrostatic adsorption and steric stabilization mechanisms in PCE-based grinding aids. Anionic functional groups, predominantly carboxylates, interact strongly with Ca^2+^ ions exposed on freshly fractured cement surfaces, enabling rapid and robust adsorption of PCE molecules during grinding [22,25,42]. When the anionic content is low, the density of adsorption sites is insufficient to ensure stable surface coverage, resulting in weak particle–polymer interactions and a limited influence on grinding efficiency.

As the anionic content increases, the adsorption strength and surface affinity of the PCE molecule are enhanced. However, if this increase occurs at the expense of nonionic segments, particularly polyethylene glycol side chains, the steric repulsion required to maintain interparticle separation becomes inadequate [25]. Under such conditions, fine particles, although strongly anchored to the surface by electrostatic interactions, can still approach each other closely and undergo re-agglomeration due to Van der Waals forces. This phenomenon limits the effectiveness of grinding by reducing the stability of the ultra-fine fractions generated.

Optimal grinding performance is therefore achieved at a balanced anionic/nonionic ratio, where strong electrostatic adsorption ensures persistent surface binding, while sufficiently developed nonionic side chains provide effective steric stabilization. This synergistic balance maximizes the generation of ultra-fine particles while simultaneously preserving PSD homogeneity and preventing excessive agglomeration [43,44].

#### 3.3.1. Effect of Anionic Charge Density and Main Chain Length of PCE-Based GAs on Grinding Performance

**Figure 7:**
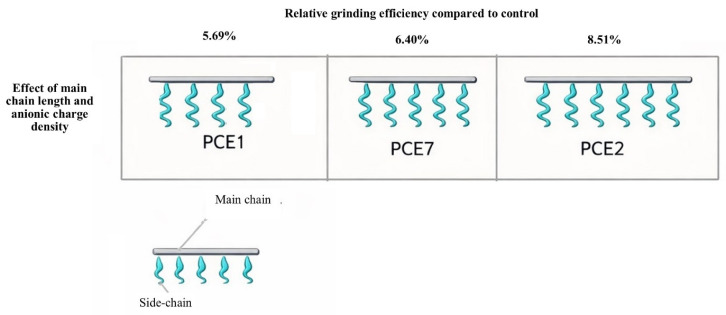
Effect of anionic charge density and main chain length on grinding performance.

The grinding performance and schematic views of PCEs, where the effect of anionic charge density and main chain length was investigated, are given in Figure 7.

In this section, the effect of main chain length and anionic charge density on grinding performance was investigated in PCE-based GAs synthesized and evaluated for grinding performance, with a fixed side chain length. A grinding efficiency of 6–8% was achieved, independent of the PCE type. According to the results obtained, an increase in the main chain length (anionic charge density) had a positive effect on grinding performance. This is thought to be due to the increased charge density of the PCE molecules, which allows them to adhere more strongly to the clinker particles.

#### 3.3.2. Effect of Side Chain Length of PCE-Based GAs on Grinding Performance

**Figure 8:**
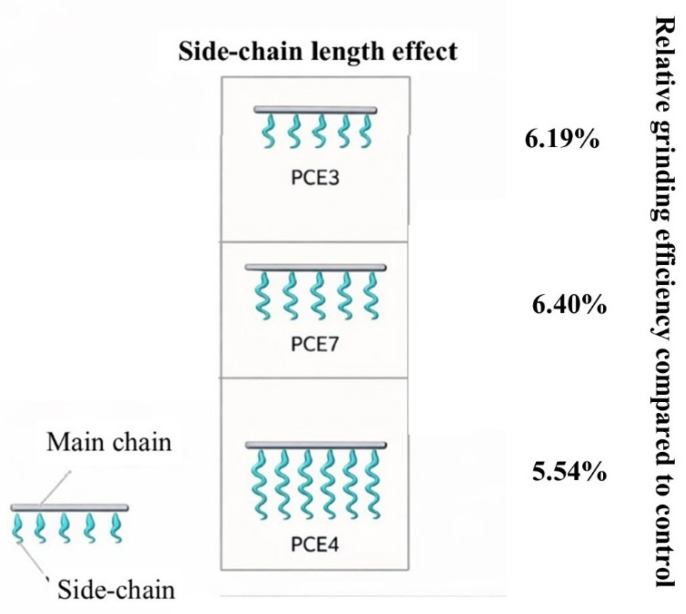
Grinding performance of PCE-based GAs with varying side chain lengths.

Figure 8 shows the grinding performance and schematic views of the PCEs whose effect of side chain length was investigated.

In this section, the effect of the side chain length of PCE-based GAs on grinding performance has been evaluated. In this context, a grinding efficiency of around 6–7% has been achieved, independent of the GA type. However, it was found that the side chain length did not significantly impact grinding performance, regardless of the type of PCE. This is thought to be because the grinding mechanism is based on neutralizing the charges on the clinker surface, and therefore, at the same anionic charge density, only the side chain length causes a significant change.

#### 3.3.3. Effect of the Main Chain/Side Chain Ratio of PCE-Based GAs on Grinding Performance

**Figure 9:**
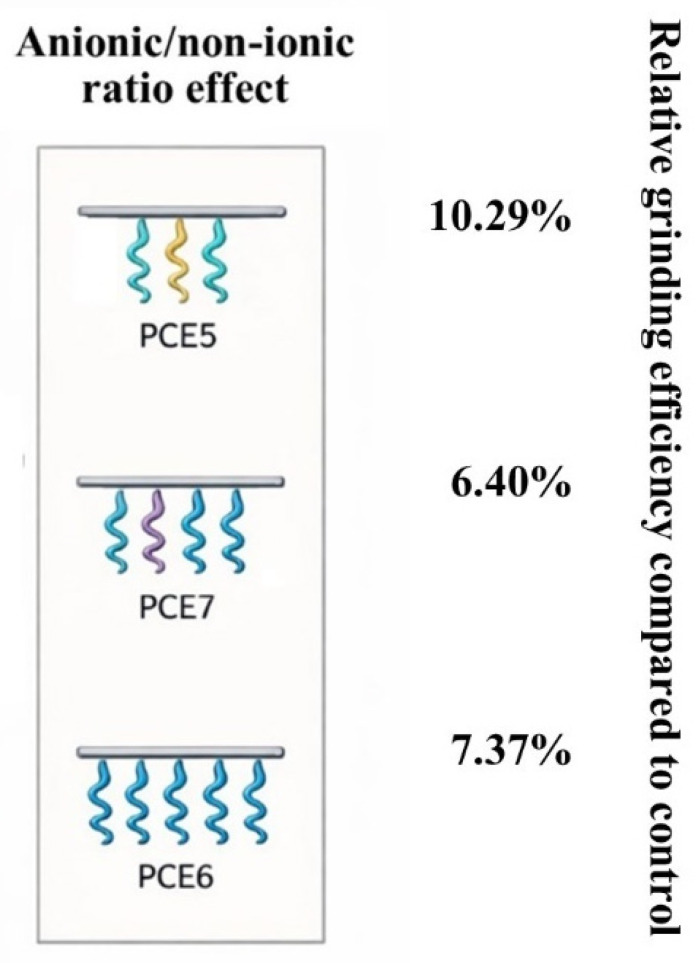
Grinding performance of PCE-based GAs with varying main/side chain ratios.

This section examines the effect of the main chain/side chain ratio of the additives on grinding performance. The grinding performances and schematic views of the PCEs are shown in Figure 9.

A grinding efficiency of 7–10% was determined in PCE-based GAs, where the main chain length and side chain length were kept constant while their ratio was varied, regardless of the type of PCE. An increase in side chain density negatively affected grinding performance. This situation is thought to be due to the negative impact on performance caused by the side chains in the additive molecule becoming entangled with each other or with different additive molecules as the side chain density increases [25,41].

**Figure 10:**
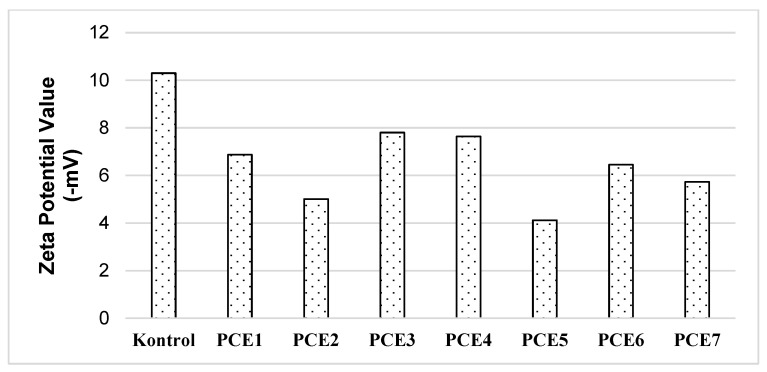
Zeta potential values of control cement without GA and cement containing GA based on PCE at a dosage of 0.05%.

**Figure 11:**
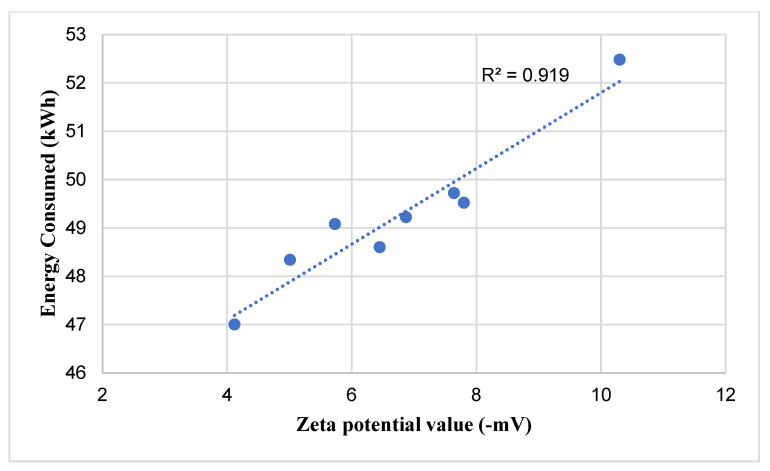
Relationship between energy expended in grinding and zeta potential values.

The Zeta potential values of the GA-free control and GA-containing cements at a dosage of 0.05% produced in the study are shown in Figure 10, and the relationship between the energy expended during grinding and the Zeta potential values is shown in Figure 11.

As shown in Figure 11, the zeta potential values of the cements produced using PCE-based grinding aids (GAs) approach zero, regardless of the GA type. A zeta potential value close to zero indicates that the electrostatic charge distribution on the cement particle surfaces is approaching equilibrium [6,42]. Furthermore, Figure 11 reveals a strong linear relationship between the energy consumed during grinding and the zeta potential values. This observation supports the hypothesis that grinding aids enhance grinding efficiency by neutralizing the surface charges of clinker particles. The highest grinding energy demand was observed for the control cement, which also exhibited the highest zeta potential value among all samples, confirming the absence of charge-neutralizing effects in systems without GA.

In conclusion, when all PCE-based grinding aids are collectively evaluated in terms of grinding efficiency, an increase in the main chain length was found to positively influence grinding performance, whereas variations in side chain length did not result in a significant change in grinding efficiency. Moreover, when the main chain and side chain lengths were kept constant, an increase in side chain density—corresponding to a higher side chain-to-main chain ratio—was observed to adversely affect grinding performance. Within this framework, PCE5 exhibited the most effective contribution to grinding efficiency among the investigated PCE-based grinding aids.

### 3.4. Cement Properties

**Figure 12:**
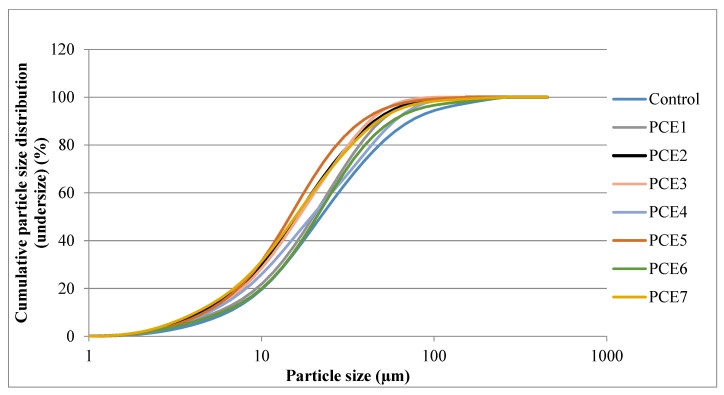
PSD curves of cements.

The particle size distributions of the cements, the d10-50-90 particle sizes, and the PSD curve slope values are given in Table 5. Additionally, the PSD curves of the cements containing 0.06% GA and those without GA are shown in Figure 12.

As shown in Table 5, the particle size distributions (PSDs) of all cements containing PCE-based grinding aids are markedly shifted toward finer fractions compared to the control sample. In the control cement, the 0–10 µm and 11–32 µm fractions account for 30.80% and 30.06%, respectively, resulting in a total fine fraction (<32 µm) of approximately 61%. In contrast, PCE-containing systems exhibit pronounced increases, particularly in the 0–10 µm and 11–32 µm size ranges, leading to the dominance of the <32 µm fraction. This behavior indicates that PCEs effectively suppress the re-agglomeration of fine particles during grinding and enhance comminution efficiency [31,32].

When the 0–10 µm fraction is specifically considered, the greatest increase relative to the control sample is observed in the PCE2 and PCE5 series, where this fraction reaches 39–42%. This finding suggests that these PCEs adsorb more effectively onto freshly generated clinker surfaces, thereby stabilizing ultra-fine particles and promoting the formation of very fine fractions [43]. Simultaneously, the proportion of coarse particles (>91 µm) decreases significantly from 6.94% in the control sample to approximately 1–3% in PCE-containing systems, demonstrating that both coarse particle breakage and suppression of coarse fractions are substantially improved.

A comprehensive evaluation of the characteristic particle diameters (D_10_, D_50_, and D_90_) further confirms the beneficial role of PCEs in grinding. In the control cement, D_10_, D_50_, and D_90_ values are 4.25 µm, 24.41 µm, and 78.15 µm, respectively. In contrast, PCE-containing systems exhibit reduced D_10_ values in the range of 2.4–3.1 µm, D_50_ values of 15.7–22.2 µm, and D_90_ values of 44.8–66.3 µm. These results demonstrate that PCEs shift not only the fine fractions but the entire PSD curve toward smaller particle sizes, thereby improving overall grinding efficiency. Notably, the minimum D_50_ and D_90_ values are observed in the PCE5 series, indicating superior grinding performance. This behavior suggests that the molecular architecture of PCE5 provides a favorable balance between electrostatic adsorption and steric hindrance effects, leading to optimal dispersion during grinding [31,32].

In contrast, certain PCE types—such as the PCE4 series—exhibit a more limited increase in fine fractions and relatively higher proportions of medium-to-coarse particles (33–60 µm). This observation highlights that not all PCE architectures exert identical effects on grinding behavior and underscores the importance of molecular design parameters. In particular, the lower performance of PCE4 in particle breakage distribution (PBD) can be attributed to its shorter main chain length (approximately 2 kg/mol), which results in reduced adsorption efficiency on clinker surfaces [22,25].

An analysis of the Rosin–Rammler distribution parameter *n* reveals that PCE-containing systems generally exhibit *n* values that are either higher than or comparable to those of the control sample, indicating not only finer but also more homogeneous PSDs. In systems where *n* ranges between 1.02 and 1.04 (e.g., PCE1-0.05 and PCE7-0.05), the PSD is confined within a narrower size range. Conversely, in systems where *n* falls below 1 (e.g., PCE2 and PCE6 series), the PSD slope becomes flatter, reflecting a broader distribution associated with increased ultra-fine fraction formation.

Overall, the incorporation of PCE-based grinding aids significantly increases the fine fraction content while markedly reducing the proportion of coarse particles. Consequently, D_10_, D_50_, and D_90_ values decrease, and the PSD becomes finer and, in most cases, more homogeneous. Nevertheless, the results clearly demonstrate that grinding performance is strongly governed by the molecular architecture of the PCE. While certain PCEs maximize ultra-fine fraction production, others primarily enhance PSD uniformity.

Consistent with the grinding efficiency analysis, the influence of PCE molecular characteristics on particle breakage distribution is summarized in the following subsection under three main categories.

#### 3.4.1. The Effect of Anionic Charge Density and Main Chain Length of PCE-Based GAs on Cement Properties

The properties of cements produced with PCEs varying in main chain length and anionic charge density are given in Table 6.

Table 6 clearly demonstrates that variations in the main chain length of PCEs significantly influence the particle size distribution (PSD) of the cement obtained after grinding. As the main chain length increases from 2 kg/mol in the PCE1 series to 4 kg/mol (PCE7) and further to 6 kg/mol (PCE2), a systematic and progressive increase in the <32 µm fraction is observed. Relative to the control sample without grinding aid, this fraction increases from approximately 112% to about 123%, indicating that longer main chains markedly promote the formation of fine particles. This behavior can be attributed to the ability of long main-chain PCE molecules to adsorb onto a larger surface area during grinding and to form a more stable and continuous dispersion layer on cement particle surfaces [17].

In parallel with the increase in main chain length, a pronounced reduction in the median particle size (D_50_) is observed. For the PCE1 series with a main chain length of 2 kg/mol, the D_50_ value is approximately 21 µm; this value decreases to around 19 µm at a main chain length of 4 kg/mol and further to about 17 µm at 6 kg/mol. These results indicate that increasing the main chain length enhances grinding efficiency by more effectively reducing friction at particle–particle contact points, thereby promoting particle fracture. Consistent with previous studies, long main-chain polymers are reported to suppress particle agglomeration by forming a more continuous adsorption film on cement surfaces, resulting in finer and more efficient grinding behavior [22,24].

An examination of the Rosin–Rammler distribution parameter (*n*) reveals a dual effect of main chain length on PSD homogeneity. In the PCE7 series with an intermediate main chain length (4 kg/mol), the increase in *n* relative to the control sample indicates a narrower and more homogeneous particle size distribution. However, when the main chain length is further increased to 6 kg/mol, the *n* value approaches that of the control sample. This suggests that excessively long main-chain PCEs may partially hinder the fracture kinetics of larger particles due to overly strong adsorption and dispersion effects, leading to a slight flattening of the PSD slope.

#### 3.4.2. The Effect of the Side Chain Length of PCE-Based GAs on Cement Properties

The properties of cements produced with PCEs with varying side chains are given in Table 7.

Table 7 demonstrates that variations in the side chain length of PCEs with a fixed main chain length of 4 kg/mol have a pronounced effect on the particle size distribution (PSD) of cement after grinding, highlighting the high sensitivity of grinding behavior to side chain architecture. Increasing the side chain length from 1 kg/mol to 2.4 kg/mol results in a notable increase in the <32 µm fraction, rising from approximately 68% to 72%. A similar trend is observed in the relative values, with the relative < 32 µm ratio increasing from about 112% to 119%. These results indicate that medium-length side chains (≈2.4 kg/mol) are particularly effective in suppressing the re-agglomeration of fine particles during grinding and in maximizing dispersion efficiency.

The influence of side chain length on the median particle size (D_50_) further supports this conclusion. The lowest D_50_ values are obtained at a side chain length of 2.4 kg/mol, ranging from approximately 18.8 to 19.1 µm. In contrast, the PCE3 series with shorter side chains (1 kg/mol) exhibits higher D_50_ values in the range of 20.7–20.9 µm, while the PCE4 series with longer side chains (3 kg/mol) shows an increase in D_50_ to approximately 21.6–22.2 µm. This trend suggests that the steric hindrance effect provided by the side chains enhances grinding efficiency up to an optimal length; however, excessively long side chains may reduce grinding efficiency due to intermolecular entanglement and less ordered adsorption on clinker surfaces [24].

An examination of the Rosin–Rammler distribution parameter (*n*) reveals that the PCE7 series with medium-length side chains attains the highest *n* values relative to the control sample, indicating a narrower and more homogeneous PSD. Although both short (1 kg/mol) and long (3 kg/mol) side chain systems also exhibit higher *n* values than the control, they remain lower than those observed for the medium-length side chains. Moreover, a slight decrease in *n* with increasing dosage is observed for these systems, suggesting that side chain length has an optimal range for achieving maximum PSD homogeneity.

#### 3.4.3. The Effect of the Anionic/Non-Ionic Ratio of PCE-Based GAs on Cement Properties

The properties of cements produced with PCEs with varying anionic/non-ionic ratio (side chain/main chain ratio) are given in Table 8.

Table 8 presents the effect of varying the anionic-to-nonionic ratio in PCE molecules while maintaining a constant main chain length of 4 kg/mol and a side chain length of 2.4 kg/mol. The results clearly demonstrate that the grinding performance of PCEs is governed not only by steric hindrance effects but also by the strength and balance of electrostatic interactions. Compared with the control sample, all PCE-containing systems exhibit substantial increases in the <32 µm fraction and pronounced reductions in D_50_ values, indicating that the anionic/nonionic balance is a key parameter controlling the extent of PSD refinement.

In the PCE5 series, characterized by a relatively low anionic/nonionic ratio (ratio = 14), the <32 µm fraction reaches values between 76.80% and 78.28%, corresponding to an increase of approximately 126–129% relative to the control. In parallel, D_50_ values decrease to around 16 µm, indicating highly efficient particle breakage and dispersion during grinding. This molecular architecture appears to provide optimal dispersion behavior, which can be attributed to the presence of a sufficient number of anionic functional groups that establish strong electrostatic interactions with Ca^2+^ ions on the cement surface, while the nonionic side chains provide effective steric stabilization of the adsorbed layer [24,42]. As a result, interparticle attractive forces are efficiently suppressed, and re-agglomeration of fine particles is minimized.

In the PCE7 series, where the anionic/nonionic ratio is increased to an intermediate level (ratio = 18), both the absolute fine fraction content and the relative < 32 µm values decrease compared to the PCE5 series. Concurrently, D_50_ values increase to the range of 18–19 µm. These results suggest that although the increased anionic content enhances molecule–surface interactions, the relative reduction in nonionic segments limits steric hindrance efficiency. This finding highlights that electrostatic repulsion alone is insufficient to achieve optimal grinding performance and that the spatial barrier provided by nonionic side chains plays a critical complementary role.

In the PCE6 series, which exhibits the highest anionic/nonionic ratio (ratio = 27), only a limited improvement in fine fraction content is observed relative to the PCE7 series, and the superior performance achieved by the PCE5 series is not reached. An evaluation of both D_50_ values and the Rosin–Rammler distribution parameter (*n*) indicates that the PSD in these systems becomes less homogeneous. Although the high anionic content promotes rapid and strong adsorption onto cement surfaces, the reduced proportion of nonionic segments limits steric stabilization at both intermolecular and particle–particle levels, leading to partial re-agglomeration of fine particles during grinding.

When the Rosin–Rammler *n* values are considered collectively, it becomes evident that a moderate anionic/nonionic balance—particularly in the PCE7 series—is more favorable for achieving PSD homogeneity. In contrast, although the PCE5 series with a lower anionic content produces the finest PSD, the relatively smaller increase in *n* values suggests a redistribution toward ultra-fine fractions accompanied by a partial flattening of the PSD slope. This observation indicates that excessively strong dispersion, while beneficial for fine particle generation, may have a dual effect by slightly reducing overall PSD uniformity.

#### 3.4.4. Powder Flow Properties

The parameters for all cements, including bulk density, apparent density, Carr index, Hausner ratio, angle of repose, and flow classes, are provided in Table 9.

#### 3.4.5. Carr Index and Hausner Ratio

The Carr index is an empirical parameter that quantifies the compressibility of a powder as a percentage, based on the difference between its bulk density and its tapped density. It is directly related to the Hausner ratio and is widely used as a rapid and practical indicator of powder flowability. A lower Carr index indicates reduced compressibility, implying weaker interparticle interactions and, consequently, improved powder flow. Similarly, the Hausner ratio provides a qualitative assessment of the flow behavior and cohesive nature of powder materials.

The improvement in the flow properties of ground cement can generally be attributed to three primary mechanisms: (i) abrasion of sharp edges and angular features of particles during grinding, resulting in smoother particle geometries; (ii) suppression of fine-particle agglomeration through the adsorption of grinding aids (GAs) on particle surfaces; and (iii) modification and, in many cases, narrowing of the particle size distribution (PSD) [8]. However, it should be noted that PSD narrowing does not universally lead to improved flowability; under certain conditions, it may increase powder cohesion and adversely affect flow behavior. Consequently, systems containing relatively larger particles may exhibit superior flowability in some cases [8].

Beyond particle size alone, the influence of PSD on powder flowability is strongly governed by particle surface morphology and the nature of interparticle forces [45]. Particles with sharp edges and rough surfaces tend to exhibit poor flowability due to enhanced mechanical interlocking and increased contact area, which promotes agglomeration. The use of grinding aids promotes particle rounding and surface smoothing, while surface adsorption of GAs effectively suppresses agglomeration. As a result, the negative effects of increased cohesion associated with narrower PSDs can be largely mitigated, allowing improved powder flow even within relatively fine and narrow particle size ranges [8,32].

**Figure 13:**
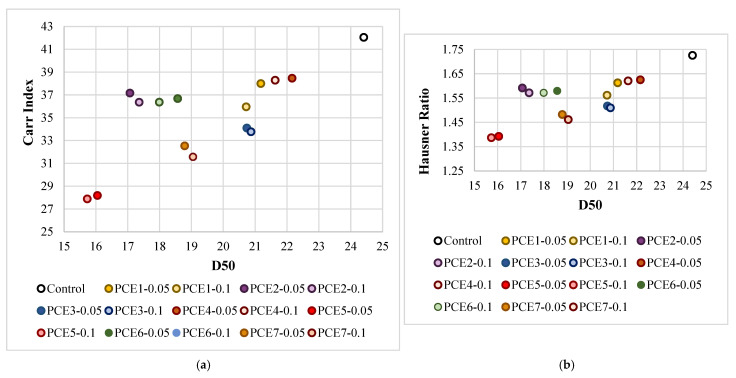
Changes in (**a**) Carr index and (**b**) Hausner ratio according to the D50 values of cements.

Within the scope of this study, the variations in Carr index and Hausner ratio were evaluated as a function of D_50_, which represents the median particle size of the cement. Accordingly, Figure 13 presents the changes in Carr index and Hausner ratio for the control cement and for cements produced in the presence of PCE-based grinding aids as a function of their D_50_ values.

As shown in Figure 13, the D_50_ values, Carr indices, and Hausner ratios of all cements produced with PCE-based grinding aids decreased compared to the control cement, regardless of additive type and dosage. This overall reduction indicates an improvement in powder flowability associated with the use of PCE-based additives.

According to the Carr index classification, the control cement was categorized as “Extremely Poor” in terms of flowability. Although the Carr indices of PCE1-0.05, PCE4-0.05, and PCE4-0.1 cements decreased relative to the control, these samples remained within the same “Extremely Poor” flow class. With the exception of PCE5, all other PCE-containing cements were classified as “Very Poor.” In contrast, the PCE5 cement exhibited the most favorable performance and was classified in the “Poor” flowability class.

The Carr indices of PCE-based cements, which were predominantly classified as “Very Poor” or occasionally “Extremely Poor,” decreased by approximately 9–20% compared to the control sample. Notably, this reduction reached 33–34% for the PCE5 series, which demonstrated the best overall flow performance. The superior performance of PCE5 is attributed to its enhanced adsorption behavior on cement particle surfaces (Figure 5 and Figure 10). Moreover, the improved flowability observed for PCE5 is associated with the increased adsorption efficiency resulting from its higher anionic/nonionic ratio [24]. These powder flowability results are fully consistent with the grinding efficiency and particle breakage distribution (PBD) findings.

In contrast, PCE4 with long side chains and PCE1 with a short main chain exhibited inferior powder flow performance compared to other additives. In the case of PCE1, the reduced adsorption efficiency is attributed to the lower density of anionic functional groups associated with its shorter main chain length [22,25,26,27]. For PCE4, the increase in side chain length is thought to reduce the effective electronegativity of the molecule, thereby weakening adsorption onto cement particles and diminishing powder flowability [22].

A similar trend is observed when the Hausner ratios are examined, which is expected given the close mathematical relationship between the Hausner ratio and the Carr index. Based on Hausner ratio classifications, the control cement, PCE1-0.05, PCE4-0.05, and PCE4-0.1 samples were again categorized as “Extremely Poor.” The PCE5 cement was classified as “Poor,” while the remaining PCE-containing cements—particularly those with higher-yielding chain architectures—were classified as “Very Poor.” Relative to the control, the Hausner ratios of most PCE-based cements decreased by approximately 6–15%, whereas a more pronounced reduction of about 19% was achieved for PCE5.

Overall, PCE5—characterized by a high anionic/nonionic ratio and optimal main chain (4 kg/mol) and side chain (2.4 kg/mol) lengths—exhibited the best powder flow performance based on both Carr index and Hausner ratio evaluations. This additive was followed by PCE7, which also possesses medium main and side chain lengths. In contrast, PCE1 with a short main chain (2 kg/mol) and PCE4 with a long side chain (3 kg/mol) showed comparatively poor flow performance among the investigated PCE-based additives.

#### 3.4.6. Angle of Repose

**Figure 14:**
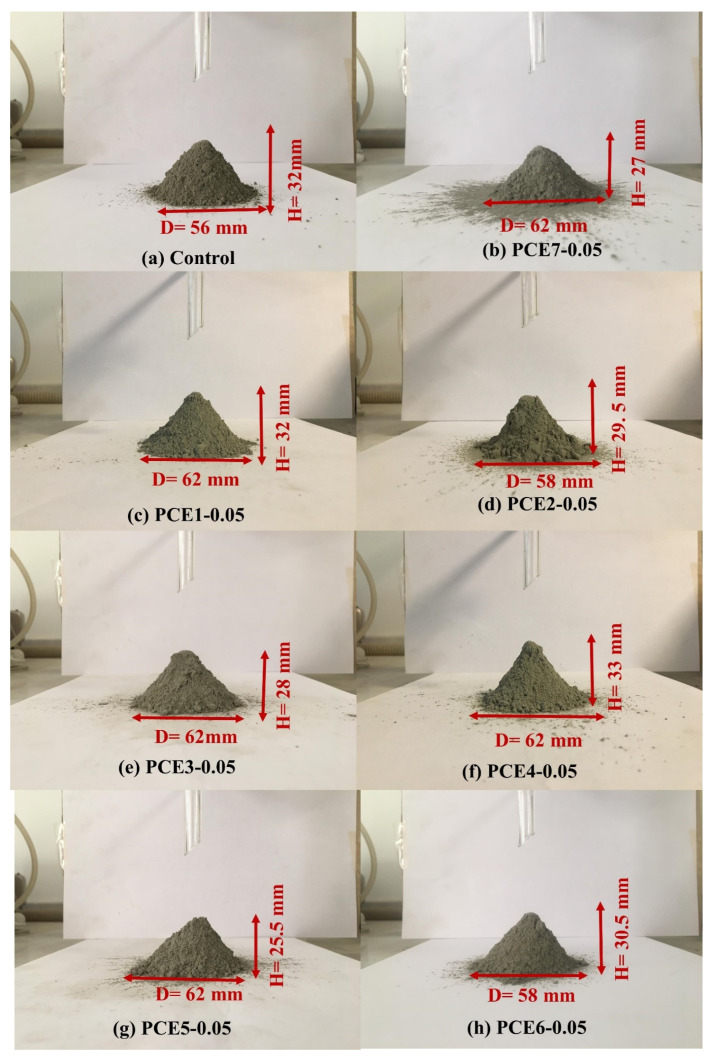
Angle of repose images of the control cement and cements containing 0.05 wt.% GA.

The angle of repose is a fundamental parameter widely used to describe the flow behavior of cement powders, as it reflects the degree of friction and interaction between particles. Higher angle of repose values indicate increased internal resistance and cohesive forces, and thus poorer powder flowability [8,46]. Images obtained from the angle of repose tests conducted on the control cement and the cements containing 0.05% PCE-based grinding aids are presented in Figure 14.

As shown in Table 9 and Figure 14, regardless of the GA type and dosage, the incorporation of PCE-based GAs resulted in a reduction in the angle of repose for all cement samples. This consistent decrease clearly indicates that the flow properties of cement powder are significantly enhanced in the presence of PCE-based GAs. The observed improvement in powder fluidity can be attributed to several micro-mechanical mechanisms that have been extensively reported in the literature [5,8,47].

At a constant Blaine specific surface area, these mechanisms can be summarized as follows: (i) abrasion of sharp edges of relatively larger particles during grinding, resulting in a more rounded and smoother surface morphology; (ii) adsorption of PCE molecules onto particle surfaces, which weakens interparticle attractive forces and suppresses the agglomeration of fine particles; and (iii) the formation of a narrower particle size distribution induced by the GA effect.

According to the flow angle classification, the control cement was categorized as “Poor.” Although the flow angles of PCE1-0.05, PCE4-0.05, PCE6-0.05, and PCE6-0.1 cements decreased compared to the control, these samples remained within the same “Poor” class (Table 9). In contrast, PCE5, PCE3-0.1, and PCE7-0.1 cements exhibited superior performance and were classified as “Fair,” representing the best flow class among the investigated samples (Table 9). The remaining cements were categorized as “Passable.

The flow angles of the highest-performing cements decreased by approximately 18–26% relative to the control, whereas reductions observed in the other cements ranged from minor values of about 2% up to 16%. When the molecular structural characteristics of the PCEs are considered, PCE5—characterized by medium main and side chain lengths and a high anionic/non-ionic ratio (i.e., a low side chain/main chain ratio)—exhibited the highest flow performance, consistent with the mechanisms discussed in previous sections.

Notably, the flow performance of the PCE3 also surpassed that of other additives at higher dosages. This behavior can be attributed to its medium main chain length (4 kg/mol) combined with a short side chain length (1 kg/mol), which enhances adsorption efficiency. The increased adsorption capacity compensates for the reduced steric dispersion associated with shorter side chains, particularly at elevated dosages [25]. Similarly, PCE7, which exhibits intermediate characteristics in terms of main chain length, side chain length, and side chain/main chain ratio, also demonstrated high flow performance at higher dosages.

### 3.5. General Assessment

Based on the experiments conducted, the best and lowest performing contributions among PCE-based SPCs are given in Table 10.

In this study, the performance of PCE-based grinding aids was not assessed using a single indicator, but rather through an integrated comparison of three key outcome groups, namely grinding efficiency, PSD refinement (especially the 0–32 µm fine fraction), and macroscopic powder flowability parameters (Carr index, Hausner ratio, and angle of repose). This holistic evaluation is essential because industrial grinding aids are expected not only to reduce the specific energy demand required to reach the target Blaine fineness, but also to improve or at least maintain the handling and flow behavior of the produced cement powder. Accordingly, the comparative summary in Table 10 highlights that PCE5 consistently exhibits the best overall performance, whereas PCE4 represents the lowest-performing architecture across all evaluated criteria. These findings indicate that the effectiveness of grinding aids is governed by the coupled mechanistic chain: PCE molecular architecture → surface interactions/agglomeration control → PSD evolution → powder flow behavior → grinding energy demand.

The superior performance of PCE5 can be mechanistically attributed to its balanced molecular architecture, which provides an optimal combination of adsorption efficiency and steric stabilization. During cement grinding, freshly fractured mineral surfaces exhibit high surface energy and strong interparticle attraction, promoting agglomeration and reducing the efficiency of comminution. In the presence of PCE5, effective adsorption onto these surfaces contributes to electrostatic charge screening/neutralization and modifies particle–particle interactions. Simultaneously, PEG side chains provide steric hindrance, preventing re-agglomeration of the newly generated fine particles. As a result, the mechanical energy applied in the ball mill is more efficiently converted into particle breakage rather than being dissipated through the formation of cohesive agglomerates. This is reflected in the significantly improved grinding efficiency and the high fraction of 0–32 µm particles obtained with PCE5. Importantly, the high fine fraction under PCE5 is not associated with deteriorated flow behavior; instead, the powder flow indices (CI/HR/AOR) indicate improved flowability, suggesting that the fine particles remain better dispersed and less cohesive. Therefore, PCE5 can be identified as the best overall additive because it simultaneously improves energy efficiency and powder handling performance—two essential requirements for practical industrial implementation.

In contrast, the inferior performance of PCE4 indicates that its architecture does not provide a sufficient level of surface stabilization under grinding conditions. A plausible explanation is that the balance between anionic charge contribution and non-ionic PEG functionality is not within the optimal range, resulting in weaker adsorption efficiency and/or limited ability to regulate surface electrostatic interactions. Consequently, cohesive interparticle forces and re-agglomeration remain dominant, reducing the effectiveness of comminution and limiting PSD refinement in the fine fraction (0–32 µm). In parallel, increased cohesion and compressibility are reflected by the poorest powder flowability indices, implying a more agglomerated, less free-flowing powder. Thus, PCE4 exhibits a dual disadvantage: both reduced grinding efficiency and degraded powder flowability, demonstrating that deviation from an optimum architecture window can negatively affect the entire process–structure–property pathway.

Overall, the comparative ranking summarized in Table 10 demonstrates that identifying the “best” grinding aid requires simultaneous consideration of grinding energy reduction, PSD refinement (including control of the fine fraction), and powder flowability. Within the investigated PCE series, PCE5 represents the most robust architecture that ensures consistent improvements across these interconnected criteria, whereas PCE4 illustrates the negative consequences of insufficient electrostatic/steric stabilization on grinding performance and powder handling. These findings support the broader conclusion that tailoring PCE molecular architecture provides a rational pathway for optimizing both comminution efficiency and macroscopic powder behavior, thereby strengthening the practical applicability of PCE-based grinding aids.

## 4. Conclusions

This study systematically investigated the influence of the molecular architecture of PCE-based GAs on grinding efficiency, cement properties, and powder flow performance when applied at two different dosages. The main findings can be summarized as follows:

- Increasing the GA dosage generally enhanced grinding efficiency and promoted the refinement of the PSD.
- At a constant side chain length, increasing the PCE main chain length significantly improved grinding efficiency and PSD refinement due to enhanced adsorption performance. However, when powder flowability was considered, cements produced with PCEs having a medium main chain length exhibited superior performance compared to those with shorter or longer main chains.
- At a constant main chain length, variations in PCE side chain length did not result in a substantial improvement in grinding efficiency or PSD refinement. These results indicate that the main chain length and anionic charge density of PCEs play a more critical role than side chain length in determining grinding performance and PSD characteristics.
- When the main and side chain lengths were kept constant, an increase in the side chain/main chain ratio (corresponding to a decrease in the anionic/nonionic ratio) led to a reduction in grinding efficiency and a deterioration in PSD. This behavior is attributed to diminished adsorption efficiency resulting from reduced electrostatic interactions.
- Considering grinding efficiency, PSD characteristics, and powder flow behavior collectively, the PCE5 additive—characterized by a medium main chain length, medium side chain length, and a low side chain/main chain ratio—was identified as the most effective GA among the investigated formulations.

## Figures and Tables

**Table 1:** Properties of clinker and gypsum.

Item (%)															
	SiO_2_	Al_2_O_3_	Fe_2_O_3_	CaO	MgO	SO_3_	Na_2_O_eq_	Cl	Combined Water (T < 230°)	Other, %	C_3_S	C_2_S	C_3_A	C_4_AF	Loss of Ignition
Clinker	21.52	5.43	3.31	65.38	1.04	0.38	0.83	0.01	-	1.58	56.51	19.06	8.79	10.07	0.52
Gypsum	4.98	1.21	0.83	28.94	0.83	39.67	0.37	-	18.93	4.24					

**Table 2:** Some characteristics of PCE-based GAs synthesized.

			PCE-Type				
Properties	PCE1	PCE2	PCE3	PCE4	PCE5	PCE6	PCE7
Solid content (%)	30.23	30.08	30.04	30.11	30.12	30.19	30.76
pH	2.87	3.26	3.16	3.44	3.54	2.94	3.97
Density (g/cm^3^)	1.090	1.080	1.090	1.080	1.078	1.082	1.100
Mw * (g/mol)	24k	70k	22k	58k	37k	69k	47k
PDI *** (Mw/Mn **)	2.3	2.1	2.3	2.0	2.1	2.2	2.1
Main chain length (g/mol)	2k	6k	4k	4k	4k	4k	4k
Side-chain length (g/mol)	2.4k	2.4k	1k	3k	2.4k	2.4k	2.4k
Side chain/main chain ratio	9	27	18	18	14	27	18

* Weight average molecular weight; ** Number average molecular weight; *** Polydispersity index.

**Table 3:** Energy values consumed during the grinding process and relative energy efficiencies.

	Blaine (cm^2^/g)	Target RPM for Blaine	Grinding Time for Target Blaine (min.)	Energy Expended (kWh)	Relative Energy Efficiency (%)
Control	3910	8750	125.0	52.48	-
PCE1-0.05	3852	8260	118.4	49.14	5.46
PCE1-0.1	3825	8120	116.7	48.80	6.93
PCE2-0.05	3818	8030	114.7	48.16	8.23
PCE2-0.1	3912	7980	114.0	47.86	8.80
PCE3-0.05	3976	8270	118.1	49.60	5.49
PCE3-0.1	3915	8180	117.0	48.90	6.90
PCE4-0.05	3842	8320	118.9	49.90	4.91
PCE4-0.1	3900	8210	117.3	49.24	6.17
PCE5-0.05	3982	7720	110.3	46.30	11.77
PCE5-0.1	3902	7980	114.0	47.86	8.80
PCE6-0.05	3810	7950	113.6	47.68	9.14
PCE6-0.1	3877	8260	118.0	49.54	5.60
PCE7-0.05	3902	8180	116.9	49.06	6.51
PCE7-0.1	3812	8200	117.1	49.18	6.29

**Table 4:** The chemical properties of PCE-based GAs and the energy efficiency they provide to the environment.

PCE	Main Chain Length (g/mol)	Side-Chain Length (g/mol)	Molecular Weight (g/mol)	Side-Chain/Main Chain Ratio	The Parameter Examined	Relative Grinding Efficiency
PCE1	2000	2400	24,000	9	Effect of anionic charge density and main chain length	5.7
PCE7	4000	2400	47,000	18		6.40
PCE2	6000	2400	70,000	27		8.51
PCE3	4000	1000	22,000	18	Effect of side-chain length	6.2
PCE7	4000	2400	47,000	18		6.40
PCE4	4000	3000	58,000	18		5.54
PCE5	4000	2400	37,000	14	Effect of side-chain/main chain ratio	10.23
PCE7	4000	2400	47,000	18		6.40
PCE6	4000	2400	69,000	27		7.37

**Table 5:** Particle size distribution and certain properties of cements.

	0–10 µm	11–32 µm	33–45 µm	46–60 µm	61–90 µm	91< µm	D10 µm ^1^	D50 µm ^2^	D90 µm ^3^	*n* ^4^
Control	30.80	30.06	14.98	9.72	7.49	6.94	4.25	24.41	78.15	0.950
PCE1-0.05	33.19	34.70	14.38	7.82	7.44	2.47	3.01	21.18	59.83	1.022
PCE1-0.1	34.04	34.48	13.54	8.16	7.22	2.56	2.94	20.72	59.62	1.002
PCE2-0.05	39.93	34.81	12.52	5.57	4.57	2.60	2.5	17.07	52.89	0.968
PCE2-0.1	39.23	35.58	11.78	5.50	5.31	2.61	2.55	17.36	54.68	0.964
PCE3-0.05	34.09	34.29	14.03	9.57	4.57	3.47	2.93	20.74	57.11	1.018
PCE3-0.1	33.65	34.79	12.47	10.25	5.65	3.20	2.97	20.87	58.42	1.004
PCE4-0.05	32.17	33.55	15.99	8.58	5.14	4.58	3.11	22.16	59.53	1.027
PCE4-0.1	32.04	35.48	13.01	8.16	7.22	4.16	3.12	21.63	66.26	0.994
PCE5-0.05	41.52	35.28	12.21	5.99	3.69	1.31	2.41	16.05	48.31	0.996
PCE5-0.1	41.78	36.50	11.91	5.43	3.32	1.06	2.39	15.73	44.81	1.017
PCE6-0.05	37.70	34.11	13.51	7.32	5.38	1.98	2.65	18.57	54.95	0.993
PCE6-0.1	38.29	35.18	12.39	6.11	6.75	1.28	2.61	17.99	55.48	0.984
PCE7-0.05	36.77	35.66	14.40	7.24	3.70	2.23	2.72	18.79	52.12	1.042
PCE7-0.1	36.15	36.15	12.08	7.21	6.06	2.35	2.77	19.05	56.91	0.994

^1,2,3^ Particle size (µm) through which 10%, 50% and 90% of cement particles pass ^4^ Slope parameter.

**Table 6:** Cement properties of PCE-based GAs according to anionic charge density.

	PCE Structural Properties			Cement Properties					
	Main Chain Length (g/mol)	Side-Chain Length (g/mol)	Main Chain/Side-Chain Ratio	0–32 µm (%)	Relative 0–32 µm (%)	d50 (µm)	Relative d50 (µm)	*n*	Relative *n*
Control				60.87	100.00	24.41	100.00	0.950	100.000
PCE1-0.05	2000	2400	9	67.89	111.53	21.18	86.77	1.022	107.594
PCE1-0.1	2000	2400	9	68.52	112.57	20.72	84.88	1.002	105.504
PCE7-0.05	4000	2400	18	72.44	119.01	18.79	76.98	1.042	109.692
PCE7-0.1	4000	2400	18	72.30	118.78	19.05	78.04	0.994	104.689
PCE2-0.05	6000	2400	27	74.74	122.79	17.07	69.93	0.968	101.874
PCE2-0.1	6000	2400	27	74.81	122.91	17.36	71.12	0.964	101.482

**Table 7:** The properties of cements produced with PCEs with varying side chains.

	PCE Structural Properties			Cement Properties					
	Main Chain Length (g/mol)	Side-Chain Length (g/mol)	Main Chain/Side-Chain Ratio	0–32 µm (%)	0–32 µm (%)	Relative 0–32 µm (%)	d50 (µm)	Relative d50 (µm)	*n*
Control				60.87	100.00	24.41	100.00	0.950	100
PCE3-0.05	4000	1000	18	68.38	112.34	20.74	84.97	1.018	107.203
PCE3-0.1	4000	1000	18	68.43	112.42	20.87	85.50	1.004	105.672
PCE7-0.05	4000	2400	18	72.44	119.01	18.79	76.98	1.042	109.692
PCE7-0.1	4000	2400	18	72.30	118.78	19.05	78.04	0.994	104.689
PCE4-0.05	4000	3000	18	65.72	107.97	22.16	90.78	1.027	108.076
PCE4-0.1	4000	3000	18	67.52	110.93	21.63	88.61	0.994	104.607

**Table 8:** The properties of cements produced with PCEs with varying anionic/non-ionic ratio (side chain/main chain ratio).

	PCE Structural Properties			Cement Properties					
	Main Chain Length (g/mol)	Side-Chain Length (g/mol)	Main Chain/Side-Chain Ratio	0–32 µm (%)	0–32 µm (%)	Relative 0–32 µm (%)	d50 (µm)	Relative d50 (µm)	*n*
Control				60.87	100.00	24.41	100.00	0.950	100.00
PCE5-0.05	4000	2400	14	76.80	126.17	16.05	65.75	0.996	104.85
PCE5-0.1	4000	2400	14	78.28	128.60	15.73	64.44	1.017	107.09
PCE7-0.05	4000	2400	18	72.44	119.01	18.79	76.98	1.042	109.69
PCE7-0.1	4000	2400	18	72.30	118.78	19.05	78.04	0.994	104.69
PCE6-0.05	4000	2400	27	71.81	117.98	18.57	76.08	0.993	104.57
PCE6-0.1	4000	2400	27	73.47	120.70	17.99	73.70	0.984	103.55

**Table 9:** The bulk density, apparent density, Carr index, Hausner ratio, angle of repose and flow class parameters of cements.

	Bulk Density (kg/m^3^)	Apparent Density (kg/m^3^)	Carr Index	Flowability Class *	Hausner Rate	Flowability Class **	Angle of Repose (°)	Flowability Class ***
Control	726.32	1253.2	42.04	Extremely Poor	1.7254	Extremely Poor	48.60	Poor
PCE1-0.05	839.43	1353.75	37.99	Extremely Poor	1.6127	Extremely Poor	46.04	Poor
PCE1-0.1	868.41	1355.85	35.95	Very Poor	1.5613	Very Poor	41.12	Passable
PCE2-0.05	874.75	1391.99	37.16	Very Poor	1.5913	Very Poor	45.45	Passable
PCE2-0.1	874.53	1374.06	36.35	Very Poor	1.5712	Very Poor	40.24	Passable
PCE3-0.05	891.81	1353.15	34.09	Very Poor	1.5173	Very Poor	42.16	Passable
PCE3-0.1	920.77	1390.09	33.76	Very Poor	1.5097	Very Poor	40.09	Fair
PCE4-0.05	834.54	1356.05	38.46	Extremely Poor	1.6249	Extremely Poor	47.48	Poor
PCE4-0.1	847.67	1373.56	38.29	Extremely Poor	1.6204	Extremely Poor	43.23	Passable
PCE5-0.05	922.33	1284.25	28.18	Poor	1.3924	Poor	39.33	Fair
PCE5-0.1	1048.45	1453.78	27.88	Poor	1.3866	Poor	36.12	Fair
PCE6-0.05	835.16	1318.89	36.68	Very Poor	1.5792	Very Poor	46.84	Poor
PCE6-0.1	886.58	1393.09	36.36	Very Poor	1.5713	Very Poor	42.21	Poor
PCE7-0.05	889.27	1317.99	32.53	Very Poor	1.4821	Very Poor	41.03	Passable
PCE7-0.1	977.67	1428.57	31.56	Very Poor	1.4612	Very Poor	39.76	Fair

* Carr Index; Excellent ≤ 10; Good: 11–15; Fair: 16–20; Passable: 21–25; Poor: 26–31; Very Poor: 32–37; Extremely Poor ˃ 38 [39]. ** Hausner Rate; Excellent: 1.0–1.11; Good: 1.12–1.18; Fair: 1.19–1.25; Passable: 1.26–1.34; Poor: 1.35–1.45; Very Poor: 1.46–1.59; Extremely Poor ˃ 1.60 [19]. *** Repose angle; Excellent: 25–30; Good: 31–35; Fair: 36–40; Passable: 41–45; Poor: 46–55; Very Poor: 56–65; Extremely Poor ˃ 66 [39].

**Table 10:** Performance comparisons of PCE-based GAs.

	Best Performance	Lowest Performance
Grinding efficiency	PCE5	PCE4
0–32 µm	PCE5	PCE4
Powder flow properties	PCE5	PCE4

## Data Availability

The original contributions presented in this study are included in the article. Further inquiries can be directed to the corresponding author.
